# UV-B Filter Octylmethoxycinnamate Alters the Vascular Contractility Patterns in Pregnant Women with Hypothyroidism

**DOI:** 10.3390/biomedicines9020115

**Published:** 2021-01-26

**Authors:** Margarida Lorigo, Carla Quintaneiro, Luiza Breitenfeld, Elisa Cairrao

**Affiliations:** 1 CICS-UBI, Health Sciences Research Centre, University of Beira Interior, 6200-506 Covilhã, Portugal; margarida.lorigo@gmail.com (M.L.); luiza@fcsaude.ubi.pt (L.B.); 2 FCS-UBI, Faculty of Health Sciences, University of Beira Interior, 6200-506 Covilhã, Portugal; 3 C4-UBI, Cloud Computing Competence Centre, University of Beira Interior, 6200-501 Covilhã, Portugal; 4 Centre for Environmental and Marine Studies (CESAM), Department of Biology, University of Aveiro, 3810-193 Aveiro, Portugal; cquintaneiro@ua.pt

**Keywords:** thyroid diseases, endocrine disruptor compound, human umbilical artery, vascular homeostasis

## Abstract

Increasing evidence relating the exposure and/or bioaccumulation of endocrine-disrupting compounds (EDCs) with cardiovascular system are arising. Octylmethoxycinnamate (OMC) is the most widely used UV-B filter and as EDC interacts with TH receptors. However, their effects on thyroid diseases during pregnancy remain unknown. The purpose of this work was to assess the short- and long-term effects of OMC on arterial tonus of pregnant women with hypothyroidism. To elucidate this, human umbilical artery (HUA) rings without endothelium were used to explore the vascular effects of OMC by arterial and cellular experiments. The binding energy and the modes of interaction of the OMC into the active center of the TSHR and THRα were analyzed by molecular docking studies. Our results indicated that OMC altered the contractility patterns of HUA contracted with serotonin, histamine and KCl, possibly due to an interference with serotonin and histamine receptors or an involvement of the Ca^2+^ channels. The molecular docking analysis show that OMC compete with T_3_ for the binding center of THRα. Taken together, these findings pointed out to alterations in HUA reactivity as result of OMC-exposure, which may be involved in the development and increased risk of cardiovascular diseases.

## 1. Introduction

The use of personal care products (PCPs) containing UV filters is a common practice in the population [1,2]. Recently, their safety has been questioned since several UV filters are classified as endocrine-disrupting compounds (EDCs). Among the main endocrine disruptive activities of EDCs the modulation of thyroid activity is highlighted [1], with studies reporting that EDCs exposure may be correlated with the incidence of thyroid diseases [3,4]. This is particularly relevant on populations more susceptible to endocrine disruption, such as pregnant women and developing fetuses [1,5,6,7,8].

The EDCs can disrupt maternal thyroid hormones (TH) production [9,10,11] inducing hypo- and hyperthyroidism. People with these pathologies have higher risk of developing cardiovascular diseases (CVDs), since small changes in TH levels can modulate vascular homeostasis [12,13]. However, the fact that the effects of UV filters acting as EDCs of the human thyroid system (and their consequences at the vascular level) during pregnancy remain unknown is a major concern.

Octylmethoxycinnamate (OMC) is probably the world’s most widely used UV-B filter [14] in the cosmetics industry [15]. In addition to its presence in hair products, lipsticks, makeup, perfumes and skin care products [16,17,18,19], the OMC is also widely found in powder samples [20], pool, tap and drinking water [21,22,23], increasing human exposure to this UV filter. Due to its high lipophilic character, low molecular weight and poor degradability, OMC is an emerging contaminant that can be bioaccumulated in several organisms [15]. Recently, this UV-B filter was banned in Hawaii owing to its toxic effects on marine ecosystems [2,24]. The toxic effects to other species have alarmed the general public about its potential to have impacts on human health when applied in human skin [2,25,26]. Indeed, the OMC can penetrate through the epidermis and dermis, and reach the systemic circulation. Consequently, OMC has been detected in urine, plasma [27], breast milk samples [19,28] and placenta [29], which raises concerns about its adverse consequences on fetal development. Several studies pointed OMC as an EDC, which have an endocrine disrupting potential by interaction with TH receptors [30,31]. In rats, it was reported that OMC-induced changes on iodine intake, on 3,5,3′Triiodothyronine (T_3_), thyroxine (T_4_) and thyroid-stimulating hormone (TSH) levels, on iodothyronine deiodinase 1 (DIO1) activity, on TSHR expression levels and on thyroid weight (see review [25]). Regarding human studies, OMC can modulate thyroid hormone receptor (THR) in human hepatocarcinoma cell lines (HepG2) [30] and is a deiodinase-disrupting chemical since it alters the expression of related genes (e.g., type II iodothyronine deiodinase (DIO2)) as reported by Song, et al. in human neuroblastoma cells [31]. However, other authors suggested that the amount of OMC absorbed by the skin after topical application does not interfere with TH homeostasis in human adults [32]. Although not entirely consensual, these results pointed an interference of OMC in the human thyroid system. However, to our knowledge no studies were performed to explore the effects of OMC in the human vasculature of pregnant women with thyroid diseases.

In this context, the purpose of the current study was to analyze the short- and long-term effects of the UV-filter OMC on arterial tonus of pregnant women with hypothyroidism. To elucidate this, human umbilical artery (HUA) rings were used in ex vivo organ bath experiments to analyze the effect and the mode of action (MOA) of OMC on the vascular contractility. Furthermore, the HUA were also used to perform cultures of vascular smooth muscle cells (SMC), which were used to evaluate cellular contractility by the planar cell surface area (PCSA) experiments to support data from organ bath. Moreover, the binding energy and the modes of interaction of the OMC into the active centre of the thyroid stimulating hormone receptor (TSHR) and thyroid hormone receptor alpha (THRα) were analyzed by molecular docking studies.

## 2. Materials and Methods

Experimental studies were performed in Health Sciences Research Centre (CICS-UBI, University of Beira Interior, Covilhã, Portugal). This work was approved by the local ethic committees (CHUCB, No.33/2018, 18 July 2018, Centro Hospitalar Universitário da Cova da Beira E.P.E., Covilhã, Portugal) and (ULS-Guarda, No.02324/2019, 27 February 2019, Unidade Local de Saúde da Guarda, Guarda, Portugal). Pregnant women gave written informed consent in accordance with the principles of the Declaration of Helsinki.

### 2.1. Sample Collection

Human umbilical cord (UC) samples were collected (*n* = 33) from normal full-term pregnancies after vaginal delivery. All donor mothers were under medication with folic acid during the first trimester of gestation or iron supplementation during the last trimester of gestation. There were two groups of pregnant women: (1) healthy donor mothers (control group) and (2) donor mothers with hypothyroidism (hypothyroidism group) treated with oral levothyroxine (Euthyrox, 2.5 µg) in generic tablets formulation, according to the standard therapy for patients with hypothyroidism [33]. Sample collection was performed after signing their written informed consent. Samples were resected from the proximal half of the UC (20 cm) and collected within 10–20 min after delivery. The tissue was immediately stored in cold (4 °C) for 4–24 h in sterile physiological saline (PSS) solution supplemented with antibiotics (penicillin, 5 U/mL, streptomycin, 5 μg/mL and amphotericin B, 12.5 ng/mL) and antiproteases (leupeptin, 0.45 mg/L; benzamidine, 26 mg/L and trypsin inhibitor, 10 mg/L) to avoid contamination and tissue degradation, respectively.

### 2.2. Preparation of HUA Rings for Vascular Reactivity Studies

Short- and long-term effects of OMC on vascular contractility patterns in pregnant women with and without hypothyroidism were investigated. The short-term effects were analyzed in HUA rings that were not incubated. To analyze the long-term effects of OMC, the HUA were preincubated 24 h with OMC at 50 μmol/L. Then, rings were used to perform vascular reactivity studies, more specifically, the arterial contractility experiments using the organ bath technique.

Human umbilical vessels were dissected as described by Cairrao, et al. [34]. Briefly, HUA were dissected free from Wharton’s jelly and cut into rings of 3–5 mm. Vascular endothelium was mechanically removed by gentle rubbing of arterial lumen with a cotton bud. The rings were mounted throughout two stainless steel wire hooks inserted through the lumen. HUA rings were suspended in the organ bath chambers LE01.004 (Letica, Madrid, Spain) filled with 20 mL of Krebs-bicarbonate solution maintained at a 37 °C and aerated with 95% O_2_ and 5% CO_2_ (pH = 7.4). Changes in isometric tension were measured in millinewton (mN) with isometric force transducers (model TRI201, Panlab SA, Madrid, Spain) coupled to an ML118/D Quad Bridge amplifier (AD Instruments, Oxford, UK) and an interface Power Lab/4SP ML750 (ADInstruments, Oxford, UK). Chart5 Power Lab software program (ADInstruments, Oxford, UK) was used to chart recording and to data acquisition. Baseline load of 20–25 mN was initially placed on the HUA rings. The rings were allowed to equilibrate for 45 min (during which the Krebs solution was replaced every 15 min) in order for the rings stretch to an optical resting tension (basal tension) of 20–25 mN.

### 2.3. Arterial Contractility Experiments

The tension recordings of HUA were performed according to our previous work [35]. Following the equilibration period and before starting the experiments, rings were transiently challenged with a supramaximal concentration of 5-HT (1 μmol/L) to assess the functional state of each vessel. Rings with a maximum contraction of <10 mN were not used [34]. Afterward, responses were stopped by washing rings with fresh Krebs solution. Vascular rings recuperated for at least 45 min, during which Krebs solution was replaced every 15 min.

In the first series of experiments, HUA rings were exposed to cumulative doses of OMC (0.001–50 μmol/L), in six steps, to evaluate their effect on basal tension of the arteries. Then, rings were contracted with 1 μmol/L of serotonin (5-HT) and the produced response was analyzed.

The next step was the analysis of OMC short-term effects. Each ring was precontracted using 5-HT (1 μmol/L), histamine (His, 10 μmol/L) or potassium chloride (KCl, 60 mmol/L). Cumulative pharmacologic concentrations (0.001–50 μmol/L) were used according to the previous studies [35,36]. Control experiments were performed using solvent (ethanol) at same % used to dissolve the OMC.

The long-term effects of 50 μmol/L of OMC were also analyzed, to observe if there is an alteration of the contractility patterns induced by exposure to OMC. In this sense, the same procedures described above to short-term effects were repeated. To avoid photodegradation of OMC [37,38] all the arterial contractility experiments were carried out with no UV light exposure.

### 2.4. Primary Cultures of HUA Smooth Muscle Cells (HUASMCs)

Cultures of HUA smooth muscle cells (HUASMCs) were obtained through explants of the umbilical artery, as previously described [35]. The SMC were grown at 37 °C in a 5% CO_2_ atmosphere in culture medium DMEM-F12 supplemented with bovine serum albumin (BSA, 0.5%), heat-inactivated fetal bovine serum (FBS, 5%), epidermal growth factor (EGF, 5 μg/mL), fibroblast growth factor (FGF, 0.5 ng/mL), heparin (2 μg/mL), insulin (5 μg/mL), penicillin (5 U/mL), streptomycin (5 μg/mL) and amphotericin B (12.5 ng/mL). The culture medium was changed three times a week. When cell growth reached a confluent culture, a trypsinization with commercial trypsin-EDTA solution (0.025%) was performed. Each culture was used until P4. HUASMC from the different passages were used to perform cellular contractility experiments (see below). Before experiments, HUASMC were placed 24 h in culture medium without FBS (FBS-free culture medium), at 37 °C in a 5% CO_2_ atmosphere, to express the required contractile phenotype [39,40].

### 2.5. Preparation of HUASMC for Vascular Reactivity Studies

Short- and long-term effects of OMC on vascular contractility patterns in pregnant women with and without hypothyroidism were investigated. Short-term effects were analyzed with non-incubated HUASMC. Long-term effects of OMC were analyzed using HUASMC that were preincubated 24 h with OMC at 50 μmol/L. Then, cells were used to perform vascular reactivity studies, namely, to evaluate cellular contractility using planar cell surface area (PCSA) technique.

HUASMC were prepared as described by our group [35,41], with some modifications as described above. Briefly, the cells were trypsinized and planted in specific Petri dishes, at 37 °C in a 5% CO_2_ atmosphere for 2 h. Then, the cells were washed with a specific saline solution (PCSA solution) and placed in an inverted fluorescence microscope (Zeiss Axio Observer Z1, Jena, Germany) with an incubation system (maintained at 37 °C) and a high-speed monochrome digital camera Axio Cam Hsm (Zeiss, Jena, Germany). Changes in the cellular area were determined in micrometres^2^ (µm^2^) by measuring the area along time through serial photographs taken before and after all experimental additions. Axion vision 4.8 software (Zeiss, Jena, Germany) was used to determine PCSA data. Measurements of the actual area were calculated using supplementary ‘‘Automatic Measurement program’’ (Zeiss). For data treatment 4–8 cells/photograph were chosen and a suitable sharp margin for its planimetric analysis was always considered.

### 2.6. Cellular Contractility Experiments

In the first series of experiments, the effect of 50 μmol/L of OMC on basal tension of HUASMC was evaluated. Then, SMC were contracted with 5-HT (1 μmol/L) and the produced effect was analyzed.

The next step was to analyze the short-term effect of OMC (50 μmol/L) on the contractile force of precontracted HUASMC, which were contracted using 5-HT (1 μmol/L) or His (10 μmol/L). After 20 min, a steady contraction was achieved, and 50 μmol/L of OMC was added to the PCSA medium to record the vascular effect.

The long-term effect of OMC 50 μmol/L was also analyzed, to observe if there is an alteration of the contractility patterns induced by exposure to OMC. In this sense, similar methodology described for short-term effects was used to evaluate the long-term effects of OMC.

The chosen concentration of OMC was 50 μmol/L since it is the concentration where the maximum effect was achieved according to organ bath data. Control experiments were always performed using solvent (ethanol) at same % used to dissolve OMC. Cellular contractility experiments were carried out with no UV light exposure to avoid OMC photodegradation.

### 2.7. Drugs, Chemicals, and Solutions

The umbilical cords samples were stored in PSS solution (pH = 7.4) with the composition: EDTA (0.50 mmol/L), KCl (5 mmol/L), HEPES (10 mmol/L), MgCl_2_ (2 mmol/L), NaHCO_3_ (10 mmol/L), KH_2_PO_4_ (0.5 mmol/L), NaH_2_PO_4_ (0.5 mmol/L), glucose (10 mmol/L), NaCl (110 mmol/L) and CaCl_2_ (0.16 mmol/L).

The composition of the Krebs’ modified solution (used in organ bath experiments) was: KCl (5.0 mmol/L), EDTA (0.03 mmol/L), MgSO_4_∙7H_2_O (1.2 mmol/L), KH_2_PO_4_ (1.2 mmol/L), ascorbic acid (0.6 mmol/L), NaCl (119 mmol/L), CaCl_2_ (0.5 mmol/L), glucose (11 mmol/L) and NaHCO_3_ (25 mmol/L) with a pH of 7.4.

The composition of the PCSA solution (used in PCSA experiments) was: NaCl (124.0 mmol/L), HEPES (5.0 mmol/L), tetraethylammonium sodium salt (TEA, 10.0 mmol/L), glucose (6.0 mmol/L), CaCl_2_ (5.0 mmol/L) and KCl (4.5 mmol/L) with a pH of 7.4.

All drugs and chemicals were purchased from Sigma-Aldrich Química (Sintra, Portugal). Stock solutions were prepared by dissolving OMC in absolute ethanol and by dissolving all the other drugs in distilled water. All of the stock solutions were stored at −20 °C. Final solutions of OMC and ethanol control were prepared daily by dilution with Krebs solution or FBS-free culture medium (according to each experiment). The final concentration of solvent never exceeded 0.05%.

### 2.8. Statistical Analysis

In arterial contractility experiments, the tension was expressed in millinewton (mN) of force elicited by HUA-rings in the presence of 5-HT, His or KCl 60 mmol/L. The relaxant responses induced by OMC were expressed as a % of reduction of the maximal contraction induced by vasoconstriction drugs. Results were expressed as mean ± standard error of the mean (S.E.M) of the number of HUA used (*n*).

Concerning cellular contractility experiments, the area achieved by HUASMC in the presence of 5-HT or His was expressed in micrometres^2^ (µm^2^). The relaxant responses induced by OMC were expressed as a % of reduction of the maximal area induced by vasoconstriction drugs. Results were expressed as mean ± standard error of the mean (S.E.M) of the number of the HUA used to obtain smooth muscle cells (*n*).

All statistical analysis was performed using SigmaStat Statistical Analysis System version 3.5 (2006) for a significance level of 0.05 and the graphic design was performed in the Software Origin 8.5.1. To analyze differences on the tension and areas induced by the contractile agents or in the % of relaxation of HUA and HUASMC, the two-way ANOVA followed by the Holm-Sidak (parametric) post-hoc test was used. This procedure was performed to compare the interactions between factors (pathological conditions and incubation with OMC) and to identify the significantly differences. When necessary, data sets were log_10_ transformed to achieve normal distribution. These criteria were checked by the Levene’s mean test and the Kolmogorov–Smirnov for homoscedasticity and normality, respectively.

### 2.9. Molecular Docking Studies

The Autodock4 program (http://autodock.scripps.edu/) was chosen to calculate the binding energy and the modes of interaction of the OMC into the active centre of the TSHR and TRHα. The 3D structural coordinates for selected target proteins TSHR and TRHα were obtained from the Protein Data Bank (https://www.rcsb.org/) and OMC was obtained from the Database of Endocrine Disrupting Chemicals and Their Toxicity profiles (DEDuCT) (https://cb.imsc.res.in/deduct/). The crystal structure of TSHR (PDB ID: 2XWT) at 1.90 Å co-complexed with its natural ligand, 2-acetamido-2-deoxy-beta-d-glucopyranose (NAG) was retrieved. Similarity, the crystal structure of TRHα (PDB ID: 2H79) at 1.87 Å co-complexed with its natural ligand, 3,5,3′Triiodothyronine (T3) was downloaded.

The Autodock Tools 1.5.6 and Quimera 1.15 software’s were used to prepare the proteins and ligands, respectively (removing water molecules, merging non-polar hydrogens atoms and adding Gasteiger partial charges) [42]. The structures of the ligands were designed in 2D using the ChemBioDraw 18.2 software and their PubChem compound identities (CIDs) and Chemical Abstracts Service Registry Number (CASRN) are presented in Table 1. To obtain 3D structures, hydrogen atoms were added, and energy minimization and geometry optimization were performed by the MMFF94 force field using the ChemBio3D 13.0 software. For the docking simulations all the structure files were saved in PDBQT format.

The Autogrid 4 was used to perform the calculations of the grid map based on the coordinates of each crystal protein structure active centre. The grid boxes with the dimensions size of 14 × 26 × 16 Å and 30 × 24 × 32 Å (along x, y, and z) with grid spacing of 0.375 Å was constructed around the active site of TSHR and TRHα, respectively.

The validation of molecular docking was achieved by RMSD values less than 2 Å and the results were subsequently confirmed using Autodock Vina. The Lamarkian genetic algorithm by Autodock 4 was used to perform all docking calculations and the remaining docking parameters were maintained as default. Finally, a total of 10 hybrid runs were obtained for each simulation and the dominating configuration of the binding complex with minimum binding energy (ΔG) was analyzed. The interactions between OMC and the selected target proteins within their active centers were visualized using the Quimera 1.15 software.

## 3. Results

### 3.1. Contractility Experiments in HUA

Direct application of different concentrations of OMC (0.001–50 μmol/L) on the control group (pregnant without pathology) and in the hypothyroidism group (pregnant with hypothyroidism) did not change their basal tension. Moreover, the incubation of 50 µmol/L of OMC did not change their basal tension. The solvent used (ethanol 0.05%) in control and hypothyroidism groups did not have an effect on the basal tension (data not shown).

#### 3.1.1. Tension Measurements of Arteries Contracted with 5-HT, His and KCl

**Figure 1:**
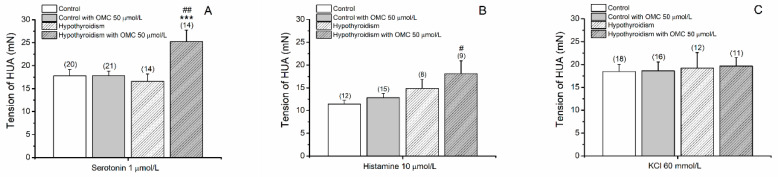
Tension (mN) (at time 15 min) of the human umbilical artery (HUA) rings without (control) and with hypothyroidism incubated with octylmethoxycinnamate (OMC, 0 and 50 μmol/L) and contracted by (**A**) serotonin (5-HT; 1 μmol/L), (**B**) histamine (His; 10 μmol/L) and (**C**) potassium chloride (KCl; 60 mmol/L). Each bar represents the mean, vertical lines the S.E.M. and the number within brackets the *n*. Asterisk * represents a significant difference versus incubation: *** *p* < 0.001, and hashtag ^#^ represents a significant difference versus hypothyroidism: ^#^ *p* < 0.05 and ^##^ *p* < 0.01; two-way ANOVA method followed by Holm–Sidak post-hoc tests.

The tension produced by 5-HT contraction in the two groups are present in Figure 1A. The results show a statistically interaction between the pathological conditions and the preincubation with OMC (*p* = 0.026). The incubation with 50 µmol/L of OMC induced a significantly higher contraction of 5-HT in the hypothyroidism group.

**Figure 2:**
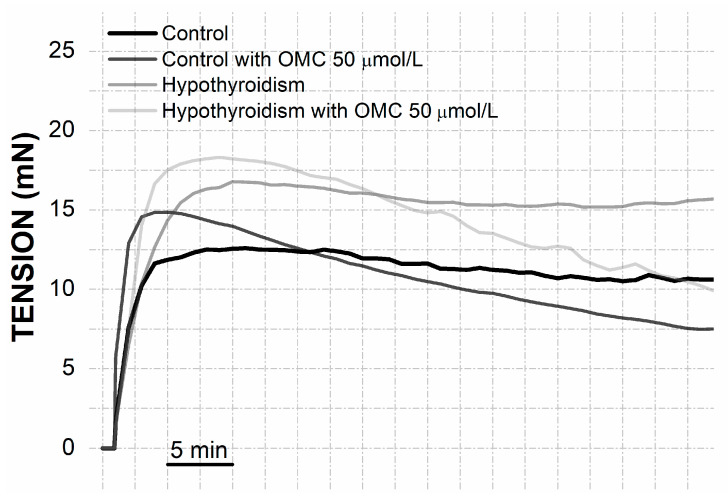
Real tension (mN) of the human umbilical artery (HUA)-rings without (control) and with hypothyroidism incubated with octylmethoxycinnamate (OMC, 0 and 50 μmol/L) and contracted by histamine (His; 10 μmol/L).

The contraction with His produced stables contractions only in arteries from control groups (Figure 2). For this reason, the tensions present in Figure 1B were attained at 15 min. The HUA from the hypothyroidism group incubated with 50 µmol/L of OMC produced a higher contraction with His (18.13 ± 2.80 mN, *p* = 0.018) when compared with the control group.

The tension produced by KCl contraction in the two groups are present in Figure 1C. The results show that the tensions produced by KCl were similar in the two groups (*p* > 0.05).

#### 3.1.2. Effects of OMC on Arteries Contracted with 5-HT

**Figure 3:**
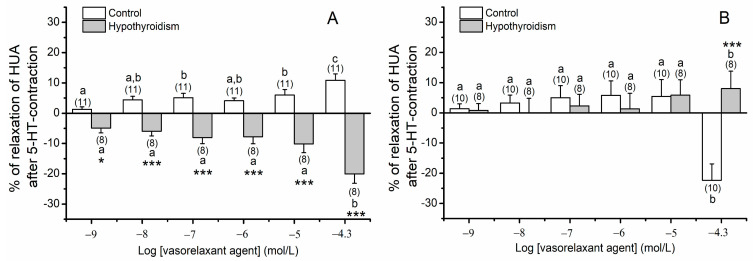
Percentage of relaxation of human umbilical artery (HUA) rings without (control) and with hypothyroidism incubated with (**A**) 0 μmol/L and (**B**) 50 μmol/L of octylmethoxycinnamate (OMC) and contracted by serotonin (5-HT; 1 μmol/L). Each bar represents the mean, vertical lines the S.E.M. and the number within brackets the *n*. Asterisk * represents a significant difference versus hypothyroidism: * *p* < 0.05 and *** *p* < 0.001, and the different letters represents significant differences between OMC concentrations (*p* < 0.05); two-way ANOVA method followed by Holm–Sidak post-hoc tests.

In Figure 3 the effects of OMC on arteries contracted with 5-HT is present. The results show a statistically significant interaction between the pathological conditions in HUA without incubation (*p* ≤ 0.001) and preincubated with 50 µmol/L of OMC (*p* = 0.001). Contrarily to the vasorelaxant effect observed for the control group, the exposure of cumulative concentrations of OMC (Figure 3A) induced a contraction effect in the hypothyroidism group. Concerning the effects of the incubation for 24 h with 50 μmol/L of OMC (Figure 3B), the results show that OMC induce a similar contractile response in the two groups, except for the highest one (OMC, 50 μmol/L) that OMC induced a small relaxation (8.03% ± 5.78%) in the hypothyroidism group, contrarily to the contraction effect observed for the control group.

#### 3.1.3. Effects of OMC on Arteries Contracted with His

**Figure 4:**
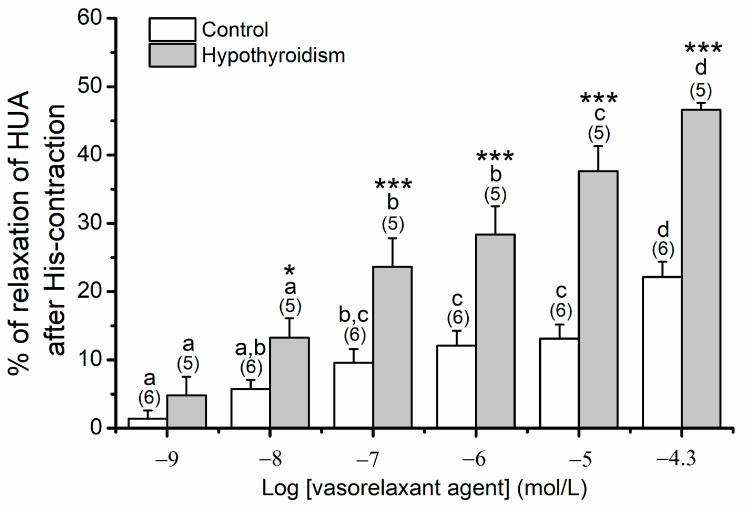
Percentage of relaxation of human umbilical artery (HUA) rings without (control) and with hypothyroidism non-incubated with octylmethoxycinnamate (OMC, 0 μmol/L) and contracted by histamine (His; 10 μmol/L). Each bar represents the mean, vertical lines the S.E.M. and the number within brackets the *n*. Asterisk * represents a significant difference versus hypothyroidism: * *p* < 0.05 and *** *p* < 0.001, and the different letters represents significant differences between OMC concentrations (*p* < 0.05); two-way ANOVA method followed by Holm–Sidak post-hoc tests.

Concerning the effects of OMC on arteries non-incubated contracted with His (Figure 4), the results show a statistically significant interaction between the pathological condition and the different concentrations of OMC of exposure (*p* ≤ 0.001). The exposure of cumulative concentrations of OMC induced a higher relaxation in the hypothyroidism group. The long-term effects of OMC on HUA contracted with His could not be assessed since stable contractions were not obtained.

#### 3.1.4. Effects of OMC on Arteries Contracted with KCl

**Figure 5:**
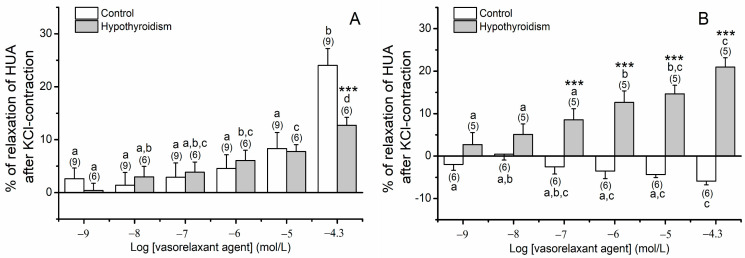
Percentage of relaxation of human umbilical artery (HUA) rings without (control) and with hypothyroidism incubated with (**A**) 0 μmol/L and (**B**) 50 μmol/L of octylmethoxycinnamate (OMC) and contracted by potassium chloride (KCl; 60 mmol/L). Each bar represents the mean, vertical lines the S.E.M. and the number within brackets the *n*. Asterisk * represents a significant difference versus hypothyroidism: *** *p* < 0.001, and the different letters represents significant differences between OMC concentrations (*p* < 0.05); two-way ANOVA method followed by Holm–Sidak post-hoc tests.

In Figure 5 the effects of OMC on arteries contracted with KCl is present. The results show a statistically significant interaction between the pathological conditions in the preincubation with 50 μmol/L of OMC (*p* ≤ 0.001). The exposure of cumulative concentrations of OMC (Figure 5A) induced a similar contractile response (relaxation) in the two groups, except for the highest concentration (OMC, 50 μmol/L) in the hypothyroidism group that induces a smaller relaxing effect. Concerning the effects of the incubation for 24 h with 50 μmol/L OMC (Figure 5B), the results show that the exposure of OMC induced a relaxation in the hypothyroidism group contrary to the observed for the control group.

### 3.2. Contractility Experiments in HUASMC

Direct application of OMC at 50 μmol/L on the control group (pregnant without pathology) and in the hypothyroidism group (pregnant with hypothyroidism) did not change their basal area. Moreover, the incubation of 50 µmol/L of OMC did not change their basal area. The solvent used (ethanol 0.05%) in control and hypothyroidism groups did not have an effect on the basal area (data not shown).

**Figure 6:**
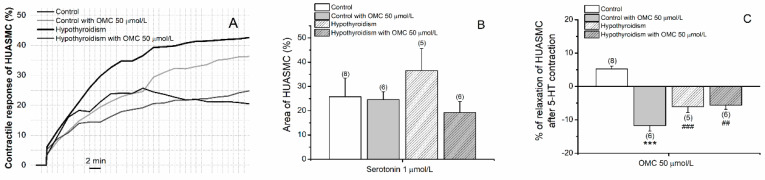
Effects of OMC on the human umbilical artery smooth muscle cells (HUASMCs) without (control) and with hypothyroidism incubated with octylmethoxycinnamate (OMC, 0 and 50 μmol/L) and contracted by serotonin (5-HT; 1 μmol/L). (**A**) Contractile response of HUASMC (%) during 40 min; (**B**) area (%) (at time 20 min) of the HUASMC and (**C**) percentage of relaxation of HUASMC after 5-HT contraction. Each bar represents the mean, vertical lines the S.E.M. and the number within brackets the *n*. Asterisk * represents a significant difference versus incubation: *** *p* < 0.001, and Hashtag ^#^ represents a significant difference versus hypothyroidism: ^##^ *p* < 0.01 and ^###^ *p* < 0.001; two-way ANOVA method followed by Holm–Sidak post-hoc tests.

In Figure 6 the effects of OMC on cells contracted with 5-HT is present. The results show that OMC induced a vasoconstriction effect in the hypothyroidism group, contrary to the observed in the control group (Figure 6C).

**Figure 7:**
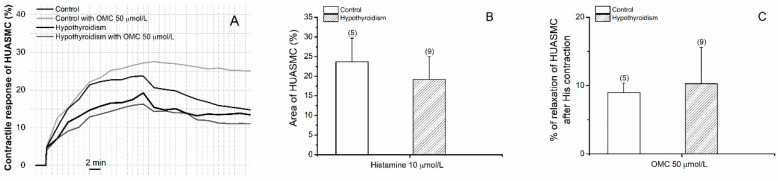
Effects of OMC on the human umbilical artery smooth muscle cells (HUASMCs) without (control) and with hypothyroidism incubated with octylmethoxycinnamate (OMC, 0 and 50 μmol/L) and contracted by histamine (His; 10 μmol/L). (**A**) Contractile response of HUASMC (%) during 40 min; (**B**) area (%) (at time 20 min) of the HUASMC and (**C**) percentage of relaxation of HUASMC after His contraction. Each bar represents the mean, vertical lines the S.E.M. and the number within brackets the *n*.

The effects of OMC on cells contracted with His are present in Figure 7. The contraction with His produced stables contractions only in HUASMC from control groups (Figure 7A). The results show that OMC in the short-term produced a vasorelaxation effect in the hypothyroidism group similarly to the control group (Figure 7C).

### 3.3. Molecular Docking Simulations

**Figure 8:**
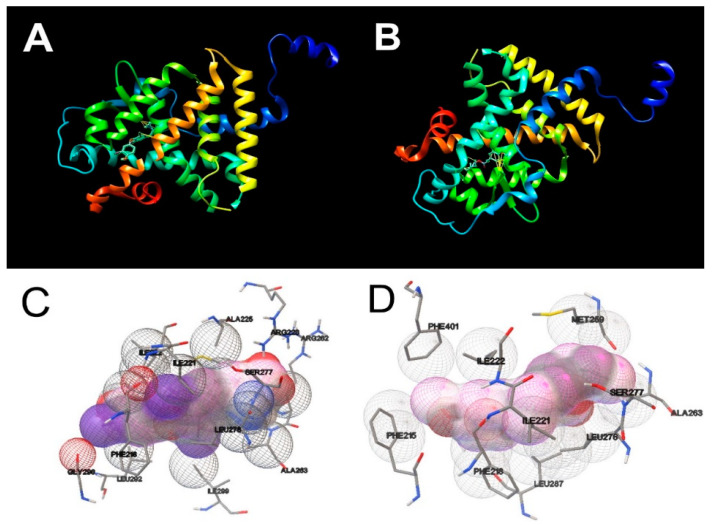
Docking views of the complex between the ligands with THRα using Quimera 1.15 software. (**A**,**B**) show the 3D representations of the interactions and preferred conformation between natural 3,5,3′Triiodothyronine (T3) and octylmethoxycinnamate (OMC), respectively, within THRα active centre. (**C**,**D**) show the interactions between natural ligand 3,5,3′Triiodothyronine (T3) and octylmethoxycinnamate (OMC), respectively, to the amino acid residues in the THRα active centre.

**Figure 9:**
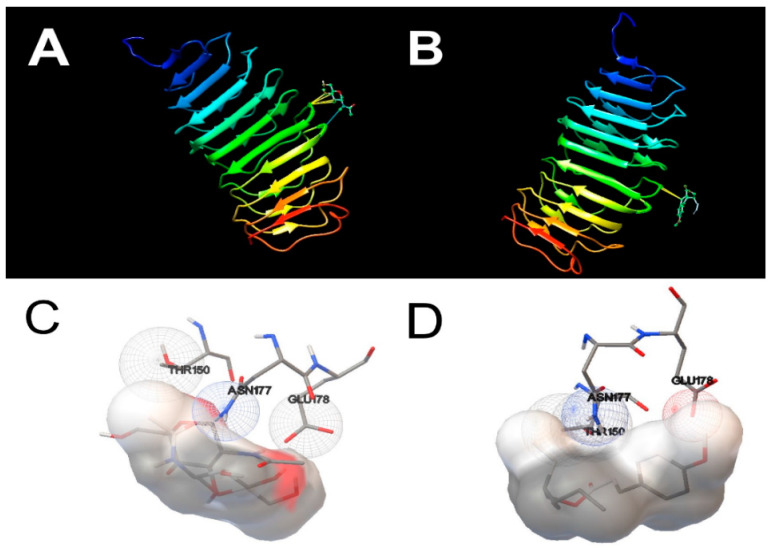
Docking views of the complex between the ligands with TSHR using Quimera 1.15 software. (**A**,**B**) show the 3D representations of the interactions and preferred conformation between natural ligand 2-acetamido-2-deoxy-beta-d-glucopyranose (NAG) and octylmethoxycinnamate (OMC), respectively, within TSHR active centre. (**C**,**D**) show the interactions between natural ligand 2-acetamido-2-deoxy-beta-d-glucopyranose (NAG) and octylmethoxycinnamate (OMC), respectively, to the amino acid residues in the TSHR active centre.

Rigid docking of OMC was carried out in the active site of the THRα and TSHR. The molecular docking results are present in Table 2 and the docking views are shown in Figure 8 and Figure 9. The molecular docking of natural ligand T_3_ with THRα shown that T_3_ is in a hydrophobic environment involving an interaction with the amino acid residues Met 259, Ser 277 and Leu 276. As shown in Figure 8A,C, T_3_ formed two H-bonds with residues Ala 180 in distances of 4.399 Å and 4.351 Å. Concerning the molecular docking of OMC with THRα, the results show that OMC is in a hydrophobic environment involving an interaction with the amino acid residues Met 259, Ser 277 and Leu 276 likewise T_3_, but also act by a hydrophobic interaction with the amino acid residues Ala 261, Ala 263, Phe 218 and Ile 222. The docking analyses show that OMC bound to the active centre of THRα with binding energy of −7.69 kcal/mol and no H-bonds were formed (Figure 8B,D).

Concerning the molecular docking results of natural ligand NAG with TSHR, the results shown that NAG is in a hydrophilic environment involving five interactions with the amino acid residue Thr 150. Moreover, NAG formed H-bonds with residue Asn 177 in a distance of 4.725 Å (see Figure 9A,C). Similarly, the molecular docking results of OMC with TSHR shown that OMC was in a hydrophilic environment, however these binding complex involved only two interactions with the amino acid residue Thr 150, and it was observed only at a greater distance (−1.5 Å). The docking analyses show that OMC bound to the active centre of TSHR with binding energy of 0.68 kcal/mol and no H-bonds were formed (Figure 9B,D).

## 4. Discussion

The proposed aim of this work was to understand how the exposure of the UV filter OMC affects the arterial tonus of pregnant women with hypothyroidism. According to Benvenga, et al. hypothyroidism is a common disorder that has a prevalence of approximately 5% and an incidence of approximately 250/100,000 per year in the adult population [43]. Oral administration of levothyroxine is the standard treatment for patients with hypothyroidism [33]. During pregnancy, supplementation with iron and folic acid is widely used and recommended in Portugal. However, several evidences indicated an interaction between iron and levothyroxine [44,45,46], which is dependent on the formulation of levothyroxine [43]. In our research, all donor mothers were under medication with folic acid during the first trimester of gestation or iron supplementation during the last trimester of gestation. Donor mothers with hypothyroidism were treated with oral levothyroxine (Euthyrox, 2.5 µg) in generic tablets formulation, according to the standard therapy for patients with hypothyroidism [33]. However, the absorption of levothyroxine appears to be reduced when the iron is present, probably due to the formation of an insoluble complex between them [46]. The simultaneous intake of levothyroxine with the oral formulation of iron leads to the need to adjust the levothyroxine dose to achieve the same levels of TH. Regarding the liquid formulation of levothyroxine, Benvenga, et al. demonstrated that it is more resistant to sequestration by calcium bicarbonate or ferrous sulphate [43] than the tablet formulation (used in this work). Thus, it would be interesting to know the levels of TH during pregnancy. Ideally, all women with hypothyroidism should be educated about the potential interaction between iron pills and levothyroxine and should be advised to avoid simultaneous intake of both [46].

Pregnant women without pathology after exposure to the UV filter OMC presented a rapid vasodilatation of HUA [35] and long-term exposure impair the vascular homeostasis of these arteries. It is known that effects of UV filters acting as EDCs of the human thyroid system (and their consequences at the vascular level) during pregnancy is a major concern as it remains unknown. Using the organ bath technique, firstly the direct effect of OMC on the basal tension of the arteries was studied. The results showed that the exposure of cumulative concentrations of OMC or the incubation of 50 µmol/L OMC did not affect the basal tension of the HUA from hypothyroidism group.

In the next step, the rapid/short and long-term effects (incubation for 24 h with 50 µmol/L) of OMC on endothelium-denuded HUA from hypothyroidism group was evaluated. The results show that OMC in the short-term induced vasoconstriction while in the long-term induced vasorelaxation of the HUA from hypothyroidism group that were precontracted with 5-HT. Nevertheless, in HUA from the control groups, OMC produced a relaxation response, which is in accordance to work demonstrated by our research group [35]. Furthermore, it was observed that the incubation with 50 µmol/L of OMC induced a significantly higher contraction of 5-HT in the hypothyroidism group. These results can be explained by the interaction observed between the pathological conditions and the preincubation with OMC but also by the vascular MOA of 5-HT as the contractile agent. Serotonin (5-HT) is the most potent vasoactive agent to contract the HUA [47] and induce vascular contraction by activation of the 5-HT_2A_, 5-HT_1B_/5-HT_1D_ and 5-HT_7_ receptors [34,48,49]. The activation of 5-HT_2A_ receptors stimulates the PLC/IP_3_ pathway and the 5-HT_1B_/5-HT_1D_ activation leads to an inhibition of adenyl cyclase. On the other hand, the activation of 5-HT_7_ receptors (Gs-protein coupled) promotes vasorelaxation by activation of adenyl [48,49,50].

The contractility experiments in HUASMC contracted with 5-HT and exposed to OMC were in accordance with the obtained in organ bath experiments, where OMC at short-term increases the area (relax) of HUASMC from the control group and decreases these areas (contraction) in HUASMC preincubated with 50 µmol/L of OMC. In relation to the experiments on HUASMC from the hypothyroidism group, OMC induced a decrease in the cell areas (relaxation), according to the organ bath data. In summary, our results suggested for the first time that the OMC could modulate the vascular homeostasis of HUA from pregnant women with hypothyroidism probably by an interference with 5-HT receptors.

In relation to the experiments regarding the effects of OMC on the vascular tone of HUA contracted with His, the results show that in the hypothyroidism group OMC in the short-term induced pronounced vasorelaxation of the HUA. In arteries from the control group, OMC also relaxes HUA according to that previously demonstrated [35]. Furthermore, it was observed that the incubation with 50 µmol/L of OMC induced a significantly higher contraction of His in the hypothyroidism group. These results can be explained by the interaction observed between the pathological conditions and the different concentrations of OMC of exposure. Concerning the long-term effects of OMC on the vascular tone of HUA contracted with His, we did not expose the HUA to the OMC since a sustained contraction of the arteries was only achieved for the control group. The fact that HUA incubated with OMC did not achieve a stable contraction with His suggests a strong interference from OMC with histamine receptors. Moreover, the pathological conditions did not alter this effect. These results might be explained by the vascular MOA of His as the contractile agent. Some authors reported that their contractile effects are due to activation of the H_1_ receptor (coupled to the Gq protein), which is present in HUA [34,49]. The His promotes vasoconstriction through the H_1_ receptor acts by PLC/IP_3_ signaling cascade and increasing [Ca^2+^]_i_ [49,51,52]. On the other hand, vasorelaxation was promoted through activation of the H_2_ receptor (Gs-protein coupled) which will activate the adenyl cyclase and decreases [Ca^2+^]_I_ [34,49,52]. Furthermore, the contractility experiments in HUASMC contracted with His and exposed to OMC were in accordance with the obtained in organ bath experiments, the HUASMC preincubated with 50 µmol/L of OMC, also did not promote a sustained contraction. Taken together, our results suggested for the first time that the OMC could modulate the vascular homeostasis of HUA from pregnant women with hypothyroidism probably by an interference with His receptors.

Concerning the experiments regarding the effects of OMC on the vascular tone of HUA contracted with KCl, the results show that OMC in the short-term induces less vasorelaxation of HUA from the hypothyroidism group than HUA from the control group. With respect to the long-term effects of OMC on vasculature of HUA, OMC induces relaxation of HUA from the hypothyroidism group contrary to that observed for the control group. These results can be explained by the interaction observed between the pathological conditions and the preincubation with OMC but also by the vascular MOA of KCl as the contractile agent. As described by our research group in previous studies, KCl contracts HUA by an influx of extracellular Ca^2+^, leading to depolarization and L-Type VOCC opening [50,53]. The contractility experiments in HUASMC for this contractile agent were not evaluated since it was impossible to perform the depolarization with isosmotic KCl (60 mmol/L) solution. Therefore, and taking into account that [Ca^2+^]_i_ is key for the contractile responses of HUA [40], our results suggest that OMC also modulates vascular homeostasis of HUA from pregnant women with hypothyroidism probably by an interference with Ca^2+^ channels. According to the literature, the dysregulation of the normal functioning of the Ca^2+^ channels (namely an increase in activity) has been associated with hypertension (HT) and other cardiovascular (CV) complications [54].

Several authors have observed an association between the CV system and the thyroid diseases, but few studies have assessed the correlation between hypothyroidism and cardiac pathologies, with the exception of atrial fibrillation and tachyarrhythmias [55,56,57,58]. The SMC, as modulators of vascular tone, are a fundamental target for the action of TH [59]. Tian, et al. suggested that TSH may have a direct vascular effect on SMC [60]. Moreover, Makino, et al. demonstrated that SMC predominantly expresses the TRHα, playing a key role in the regulation of the vascular tone [61]. According to the literature, the TH (mainly T_3_) may act directly in the vascular smooth muscle cells inducing relaxation [62,63,64]. Ojamaa, et al. demonstrated that this vasorelaxation occurs within 10 min after T_3_ is bound to specific binding sites in the SMC. The authors were unable to detect increases in cGMP levels in endothelial cells (EC) after T_3_ stimulation, which is suggestive that NO was not produced in these cells. Therefore, these results suggested that T_3_ acts by a non-genomic mechanism and endothelium-independent [65]. Moreover, Fukuyama, et al. suggested that the mechanism for this vasorelaxant effect may be due to decreasing the expression of angiotensin II type 1 receptors by T_3_, and thereby reduces the contractile response to angiotensin II [66]. According to these authors, Carrillo-Sepulveda, et al. also demonstrated that T_3_ causes nitric oxide (NO)-dependent rapid relaxation of vascular SMC by activation of PI3K/Akt-mediated endothelial nitric oxide synthase signaling pathway [67]. Consequently, recent epidemiological studies have shown that patients with hypothyroidism or with subclinical hypothyroidism may develop increased diastolic blood pressure, probably via decreased endothelium-mediated relaxation and vascular compliance [55,62,68]. In this sense, some studies suggested that this vasodilation is a result of the T_3_ effects on the PI3K/Akt pathway mediated by non-genomic and genomic actions [69,70]. Interesting, Napoli, et al. reported that TSH (thyroid stimulating hormone) can promote endothelial mediated vasodilation of conduit arteries, independent of systemic hemodynamic changes [71]. Moreover, Zwaveling, et al. suggested that relaxation induced by TH may be the result of a joint effect between EC and SMC. Briefly, TH can act directly on SMC and EC, and these last cells can induce indirect effects on SMC [72]. Taken together, these data indicate that TH exerts vasorelaxant effects at the vascular level.

The TH plays a fundamental role in CV homeostasis and vascular remodeling [12,73]; myocardial and vascular endothelial tissues have thyroid receptors and even subtle changes in circulating TH concentrations can adversely influence the CV system [12]. However, specific links and mechanisms between an altered thyroid metabolism and CV diseases, during the progression of disease from organ specific to systemic disorder, are not known and need to be established. Furthermore, current human exposure to ubiquitous chemicals (from cosmetics to environment), which might act as thyroid disrupting chemicals by disrupting thyroid homeostasis can contribute to the development and increased risk of CV diseases [74].

Organic UV filters are emerging contaminants present ubiquitously in the environment. Human exposure to these EDCs is a global concern due to their adverse effects on human hormone systems [75,76] and an important target for thyroid disruption [76] namely in vulnerable populations such as pregnant women [1,5,6,7,8,9].

The EDCs affect the normal functioning of TH at a molecular level by different mechanisms, including hormone receptor proteins. The THR is the nuclear receptor responsible for thyroid signaling [76] and TSHR is the primary regulator of thyrocyte function that regulates the levels of circulating T_3_ and T_4_ hormones [77]. Therefore, both TRHα and TSHR are proteins involved in thyroid physiology that can be potential targets for EDCs.

One of the main research difficulties is to clarify whether the mode of action of EDCs with receptors is due to an endocrine hormone mimicking action or an interference with the signal transduction process [78]. In this way, molecular docking analysis helped us by providing some information on structure–activity relationships for thyroid receptors by OMC. The results of our study revealed that OMC successfully binds to the active centre of TRHα, but the same was not observed for TSHR. Interactions of the OMC with more amino acid residues were found with TRHα, when compared with the native ligand, the hormone T_3_. Comparing with the natural TSHR ligand (NAG ligand), the OMC binding involved fewer interactions with amino acid residues, and a greater distance was required for these interactions to be observed. Therefore, our results indicate that OMC has a higher binding affinity for TRHα than for TSHR, which is also confirmed by the binding energies. Typically, lower binding energies indicate a higher possibility of stable binding [75], and the OMC binding energy value for the TRHα receptor (−7.69 kcal/mol) has a more negative value than for the TSHR (0.68 kcal/mol). Moreover, the OMC is a hydrophobic compound [79], so it is not surprising that its binding to the TRHα receptor occurred in the internal hydrophobic cavity. Contrary, the OMC binding to TSHR in a hydrophilic environment, which supports the weak affinity of this UV filter to TSHR. Thus, our results seem to indicate that OMC has the potential to interfere with the binding of T_3_ to TRHα. One of the reasons for the greater potential of OMC binding to the TRHα receptor compared to TSHR may be due to the structural similarity shared between OMC and T_3_ (both have a double ring chemical structure). Therefore, it is expected that OMC will imitate T_3_ and compete for the active centre of TRHα. Although molecular docking simulation cannot provide an absolute or precise mode of action of OMC, our results suggested that OMC seems to disrupt TH signaling pathways, which have implications at the genomic and non-genomic level, supporting the contractility data obtained.

According to Couderq, et al. [80] assessing the effects of a compound’s endocrine disruption is a challenge due to (i) the existence of non-monotonic responses, which leads to questioning where the “safe” threshold doses are determined; (ii) the endocrine system is an integrative and complex system that may involve different hormonal pathways; (iii) the existence of critical stages of development such as pregnancy, which are more vulnerable to exposure to EDC and (iv) the effects of exposure may occur at a later stage of development or even extend to future generations. This concept was introduced by the Developmental Origins of Health and Disease theory (DOHaD), which highlights the importance that EDCs can play in fetal programming [81]. Fetal programming is understood as the “result of epigenetic changes that occurs in response to various stimuli that come from the environment that can affect the life and health of the baby even in adulthood” [82]. According to previous studies, the placenta is not fully effective against these chemical compounds, and even its exposure in pregnant women has been associated with the entry of these compounds into fetal circulation [83,84,85]. Regarding the OMC, it is described that this UV-B filter can penetrate through the epidermis and dermis and reach the systemic circulation. Consequently, OMC has been detected in urine, plasma [27], breast milk samples [19,28] and placenta [29]. Thus, our results may indicate that the OMC effects may be remarkable in the future generation, since the developing fetus is more sensitive to EDCs than a human adult [81]. Based on the fetal programming hypothesis, pregnancies affected by diseases such as gestational diabetes appear to be associated with endothelial dysfunction of the human umbilical vein (HUV) [86]. Moreover, in HUVEC from women’s with pre-eclampsia presented functional abnormalities of calcium handling and NO production [87]. More recently, sex differences in HUVEC have also been reported. However, this sex difference was not observed in HUASMC [82]. Therefore, taken together, our findings suggested that alterations in maternal reactivity vasculature as a result of OMC exposure might reflect long-term “programming” of the fetal cardiovascular system. According to this concept, the exposure to OMC in the prenatal stage may be involved in the development and increased risk of cardiovascular diseases.

In summary, our results suggested that OMC alters the vascular contractility patterns in pregnant women with hypothyroidism. Pronounced vasorelaxation or vasoconstriction as the ones obtained in this study as a response to OMC might be harmful to the CV system of pregnant women. However, further studies are needed to unravel the vascular MOA of OMC, which may also be related with 5-HT and His receptors or the Ca^2+^ and/or K^+^ channels. The activation of ion channels could also be an explanation for the exaggerated relaxation observed in HUA from the hypothyroidism group contracted with His and explain the non-sustained contractions obtained. According to Gokina and Bevan working with rabbit cerebral arteries, sustained contractions of His may be due to an increase in Ca^2+^ currents through VOCC, sensitization of the contractile apparatus and the non-selective cationic channels [88]. Therefore, these results are in accordance with other investigations reporting that long-term exposure to EDCs can be the inductor of CV complications [1,11,89,90,91] and are extremely important at a physiological and pharmacological level to improve thyroid and CV maternofetal health.

## 5. Conclusions

To the best of our knowledge, this study is the first report to rapid- and long-term effects of the UV filter octylmethoxycinnamate (OMC) on vasculature from pregnant women with hypothyroidism. Our results indicated that OMC altered the contractility patterns of HUA contracted with serotonin, histamine and KCl, possibly due to interference with serotonin and histamine receptors or an involvement of the Ca^2+^ channels. The molecular docking analysis show that OMC compete with T_3_ for the binding centre of THRα. Taken together, these findings pointed out alterations in HUA reactivity as a result of OMC-exposure, which may be involved in the development and increased risk of cardiovascular diseases.

In conclusion, this work represents a new and promising research field that remains practically unexplored, and therefore requires further investigations. Given the ubiquity of this UV filter in the environment and its potentially adverse effects on human health, studying human exposure to OMC may lead to a better understanding of the role of this EDC in cardiovascular diseases. Furthermore, due to the close relationship between the CV and thyroid systems, it highlights the need to identify the molecular pathways involved in the effects of EDCs for better prevention and treatment of CVD in pregnant women with hypothyroidism.

## Figures and Tables

**Table 1:** Nomenclature, commonly used abbreviations, PubChem IDs and the Chemical Abstracts Service Registry Number (CASRN) of ligands for molecular docking studies with the thyroid stimulating hormone receptor (TSHR) and thyroid hormone receptor alpha (TRHα).

S. No	Name	Abbreviation	PubChem ID	CASRN
1	3,5,3′Triiodothyronine	T_3_	5920	6893-02-3
2	2-acetamido-2-deoxy-beta-d-glucopyranose	NAG	24139	7512-17-6
3	octylmethoxycinnamate	OMC	5355130	5466-77-3

**Table 2:** Binding energies of ligands T3, NAG and OMC (1–10) calculated from molecular docking studies.

Compound	TRHα	TSH
T_3_	−10.80	-
NAG	-	−1.61
OMC_1	−7.69	0.68
OMC_2	−7.67	0.69
OMC_3	−7.65	0.73
OMC_4	−7.63	0.96
OMC_5	−7.60	1.08
OMC_6	−7.56	1.16
OMC_7	−6.92	1.01
OMC_8	−7.35	1.11
OMC_9	−7.16	1.30
OMC_10	−6.71	1.23

## Data Availability

Data is contained within the article.
